# The Anabolic Response to Plant-Based Protein Ingestion

**DOI:** 10.1007/s40279-021-01540-8

**Published:** 2021-09-13

**Authors:** Philippe J. M. Pinckaers, Jorn Trommelen, Tim Snijders, Luc J. C. van Loon

**Affiliations:** Department of Human Biology, School of Nutrition and Translational Research in Metabolism (NUTRIM), Maastricht University Medical Centre+, P.O. Box 616, 6200 MD Maastricht, The Netherlands

## Abstract

There is a global trend of an increased interest in plant-based diets. This includes an increase in the consumption of plant-based proteins at the expense of animal-based proteins. Plant-derived proteins are now also frequently applied in sports nutrition. So far, we have learned that the ingestion of plant-derived proteins, such as soy and wheat protein, result in lower post-prandial muscle protein synthesis responses when compared with the ingestion of an equivalent amount of animal-based protein. The lesser anabolic properties of plant-based versus animal-derived proteins may be attributed to differences in their protein digestion and amino acid absorption kinetics, as well as to differences in amino acid composition between these protein sources. Most plant-based proteins have a low essential amino acid content and are often deficient in one or more specific amino acids, such as lysine and methionine. However, there are large differences in amino acid composition between various plant-derived proteins or plant-based protein sources. So far, only a few studies have directly compared the muscle protein synthetic response following the ingestion of a plant-derived protein versus a high(er) quality animal-derived protein. The proposed lower anabolic properties of plant- versus animal-derived proteins may be compensated for by (i) consuming a greater amount of the plant-derived protein or plant-based protein source to compensate for the lesser quality; (ii) using specific blends of plant-based proteins to create a more balanced amino acid profile; (iii) fortifying the plant-based protein (source) with the specific free amino acid(s) that is (are) deficient. Clinical studies are warranted to assess the anabolic properties of the various plant-derived proteins and their protein sources in vivo in humans and to identify the factors that may or may not compromise the capacity to stimulate post-prandial muscle protein synthesis rates. Such work is needed to determine whether the transition towards a more plant-based diet is accompanied by a transition towards greater dietary protein intake requirements.

## Key Points

**Table:** 

It has been suggested that the muscle protein synthetic response to the ingestion of a single bolus of plant-derived protein is less robust when compared with the response following ingestion of an equivalent amount of animal-derived protein. However, this comparison remains limited to a few plant-derived proteins.
Most plant-derived proteins have a lower essential amino acid content when compared with animal-derived proteins, and many are deficient in specific amino acids such as lysine or methionine. However, there is considerable variation in amino acid composition between various plant-based proteins.
The muscle protein synthetic response to plant-derived protein ingestion may be improved by increasing the amount of protein ingested. In addition, it has been speculated that consuming blends of different plant-derived proteins or consuming plant-derived proteins fortified with the deficient (free) amino acid(s) increases the post-prandial muscle protein synthetic response.

## Introduction

A single exercise session increases muscle protein synthesis rates, and to a lesser extent, muscle protein breakdown rates [1]. However, net muscle protein balance does not become positive unless exogenous amino acids are provided [2]. Dietary protein ingestion increases muscle protein synthesis rates at rest [3–5] and further increases muscle protein synthesis rates during recovery from exercise [2, 6, 7]. Previous work has shown that besides the amount of protein [8–11], the digestion and absorption kinetics [12] and amino acid composition of a protein (source) [13, 14] largely determine the muscle protein synthetic response to feeding. The muscle protein synthetic response to protein ingestion can, therefore, vary substantially between different dietary protein sources [13–17]. The differential muscle protein synthetic response to feeding is largely dependent on the post-prandial rise in plasma essential amino acid concentrations [5], with plasma leucine concentrations being of particular importance [18–24]. The post-prandial rise in circulating amino acids and the subsequent increase in muscle protein synthesis rate are regulated on various levels, ranging from dietary protein digestion, amino acid absorption, splanchnic amino acid sequestration, post-prandial tissue perfusion, uptake of amino acids by the muscle, and the activation of the muscle protein synthetic machinery [4, 25]. To date, most studies have focused on assessing the post-prandial muscle protein synthetic response to dairy protein [15, 17, 21, 26–31] and meat [10, 32–34] ingestion. The substantial increase in muscle protein synthesis rates observed following ingestion of these proteins or protein sources has been attributed to the rapid post-prandial rise in circulating plasma essential amino acid concentrations.

With the global population projected to reach approximately 9.6 billion by 2050, the production of sufficient amounts of conventional animal-based, protein-dense foods to meet global dietary protein demands may no longer be desired or feasible. Affluent Western societies show a strong trend in the transition towards a more plant-based diet [35]. This includes an increase in the consumption of plant-based proteins at the expense of animal-based proteins. Although the current market already offers a wide selection of plant-derived proteins and plant-based protein sources, there is a paucity of studies that have assessed the bio-availability and anabolic properties of plant-based proteins [13, 14, 16, 36–38]. Some [14, 16, 36], but not all [13, 37, 38] of these studies show that the ingestion of plant-derived proteins, such as soy and wheat protein, results in a lower muscle protein synthetic response when compared with the ingestion of an equivalent amount of animal-derived protein. Consequently, plant-based proteins are typically considered to have lesser anabolic properties. However, this concept is based on a limited number of comparisons and may not translate to all plant-based protein sources. The proposed lesser anabolic properties of plant- versus animal-based proteins have been attributed to differences in their protein digestion and amino acid absorption kinetics, as well as to differences in amino acid composition between these proteins. Previously, we reported substantial differences in amino acid composition between various plant-based protein sources [39]. Although the amino acid composition can be quite variable between different plant-based proteins, most plant-based proteins are relatively low in essential amino acid content and are often deficient in one or more specific amino acid, such as leucine, lysine, and/or methionine [39]. So far, only a few studies have directly compared the muscle protein synthetic response following the ingestion of a plant-derived protein versus a high(er) quality animal-derived protein [13, 14, 16, 36–38]. Furthermore, even less is known about the different strategies that can be applied to improve the anabolic properties of plant-based proteins.

The purpose of this review is to provide an updated overview on the bio-availability and anabolic properties of plant-based proteins in vivo in humans. We will discuss different strategies that can be applied to compensate for the lesser quality of plant-based proteins and, as such, to increase post-prandial muscle protein synthesis rates. We will discuss the need to advance nutrition research by extending studies from merely comparing post-prandial muscle protein synthesis rates following the ingestion of plant- versus animal-derived protein isolates or concentrates to assessing the impact of ingesting whole foods and mixed meals on post-prandial muscle protein synthesis. Finally, we will discuss the current beliefs regarding the use of plant-based proteins in the field of sports nutrition, and provide examples of other alternative protein sources that can be applied to support muscle conditioning in the future.

## Protein Digestion and Amino Acid Absorption

Following food ingestion, dietary protein needs to be digested and absorbed for the amino acids to become available in the circulation, where they can modulate muscle tissue protein synthesis and breakdown rates. Protein digestion occurs in the mouth, stomach, and small intestine, where protein undergoes mechanical and chemical breakdown into smaller constituents [40]. When amino acids are subsequently taken up from the gastrointestinal lumen they are considered to be absorbed. A substantial part of the absorbed amino acids will be retained and metabolised in the splanchnic region, but the majority will be released in the circulation, after which they become available for uptake into peripheral tissues. The quantitative assessment of protein digestibility, absorbability, splanchnic extraction, and amino acid release in the circulation is complex and only a few studies have tried to quantify post-prandial protein handling in vivo in humans [4]. Studies have reported substantial differences in protein digestion and amino absorption kinetics following ingestion of different proteins and protein sources. In general, plant-based whole foods have a lower absorbability when compared with animal-based whole foods. For example, recent data in humans have shown that ~ 85–95% of the protein in egg whites, whole eggs, and chicken is absorbed, compared with only ~ 50–75% of the protein in chickpeas, mung beans, and yellow peas [41, 42]. The lower absorbability of plant-based proteins may be attributed to anti-nutritional factors in plant-based protein sources, such as fibre and polyphenolic tannins [43]. This seems to be supported by the observation that dehulling mung beans increases their protein absorbability by ~ 10% [44]. When a plant-based protein is extracted and purified from anti-nutritional factors to produce a plant-derived protein isolate or concentrate, the subsequent protein absorbability typically reaches similar levels as those observed for conventional animal-based protein sources [45]. This implies that the low absorbability of plant-based protein sources is not an inherent property of a plant-based protein per se, but simply a result of the whole-food matrix of the protein source.

Protein absorbability has long been recognised as a crucial component of the nutritional quality of a protein source [46]. Currently, the Food and Agriculture Organization of the United Nations (FAO) and World Health Organization (WHO) recommend the Digestible Indispensable Amino Acid Score (DIAAS) to quantify dietary protein quality [47]. The DIAAS of a protein is based on its capacity to meet the requirements of each indispensable amino acid, which is reflected by the amino acid profile and absorbability of each individual indispensable amino acid. However, a limitation of the DIAAS score is that it only accounts for overall protein absorbability (cumulative absorption) and not for amino acid absorption kinetics (the rate at which amino acids are being absorbed). Several studies suggest that a more rapid rate of amino acid absorption is an independent factor that modulates the muscle protein synthetic response to feeding [17, 48–50], although such association is not always observed [51, 52]. There are few data available on the amino acid absorption kinetics following the ingestion of plant-based protein sources or plant-derived protein isolates or concentrates. With regards to the post-prandial rise in circulating amino acid concentrations as a proxy for protein digestion and amino acid absorption, data seem to suggest that plant-derived protein isolates or concentrates are rapidly digestible [13, 16, 38, 53, 54] and do not seem to differ substantially from most animal-derived proteins or protein sources. It is more than likely that the anti-nutritional factors in plant-based protein sources (whole foods) not only compromise overall protein absorbability, but also attenuate the post-prandial rise in amino acid absorption rates. Because of the apparent differences in protein absorbability and protein digestion and amino acid absorption kinetics, we need to be careful when referring to plant-based proteins to specify them as either plant-based protein sources or rather as plant-derived protein isolates or concentrates.

## Amino Acid Composition of Protein

**Fig. 1:**
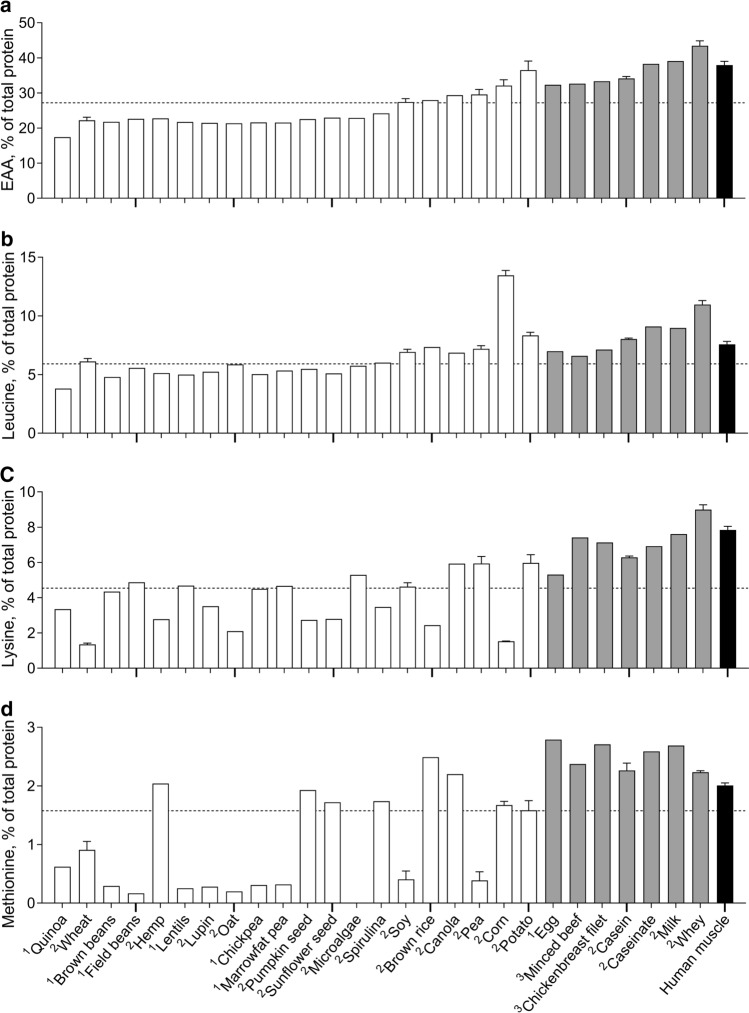
Essential amino acid (EAA, Panel **a**), leucine (Panel **b**), lysine (Panel **c**), and methionine (Panel **d**) contents (expressed as % of total protein) of various dietary protein sources and human skeletal muscle protein. White bars represent plant-based protein sources, grey bars represent animal-derived protein sources, and the black bar represents human skeletal muscle protein. Dashed line represents the amino acid requirements for adults (WHO/FAO/UNU Expert Consultation 2007 [58]). Note: EAA is the sum of histidine, isoleucine, leucine, lysine, methionine, phenylalanine, threonine, and valine, as tryptophan was not measured. Values obtained from multiple products are expressed as mean (± SEM). This figure represents an extension from data previously presented by Gorissen et al. 2018 [39], assessed using the same method. ^1^ Flour, ^2^ Protein concentrate/isolate, ^3^ Freeze-dried raw product

Following dietary protein digestion and amino acid absorption, a large proportion of the dietary protein-derived amino acids is released in the circulation. The post-prandial increase in plasma amino acid concentration activates the protein synthetic machinery in skeletal muscle tissue while also providing the necessary precursors to allow muscle protein synthesis rates to increase [5, 7, 55]. The essential amino acids are considered to be mainly responsible for the post-prandial stimulation of muscle protein synthesis [55]. In agreement, a dose-dependent relationship has been reported between the amount of essential amino acids ingested and the post-prandial muscle protein synthetic response [56]. Consequently, proteins with high(er) essential amino acid contents are generally considered high(er) quality proteins and are also more likely to (strongly) stimulate post-prandial muscle protein synthesis. Previously, we have shown that the essential amino acid contents of plant-based proteins are generally lower when compared with animal-derived proteins [39, 57]. In the current review, we included an extended overview of the amino acid composition of a wide variety of protein (sources) we have analysed (Fig. 1a). However, there are also plant-based proteins (such as soy, brown rice, canola, pea, corn and potato protein) that have relatively high essential amino acid content, meeting the requirements recommended by the WHO/FAO/UNU (United Nations University) [58]. In fact, the essential amino acid contents of canola- (29%), pea- (30%), corn- (32%) and potato- (37%) derived protein are comparable or even greater than casein (34%) or egg (32%) protein [39]. Therefore, certain plant-based proteins could, in theory, provide sufficient essential amino acids to allow a robust post-prandial increase in skeletal muscle protein synthesis rate.

Among all of the essential amino acids, leucine represents the amino acid with the strongest anabolic properties. Leucine is sensed by sestrin2, which promotes translocation of mammalian target of rapamycin complex 1 (mTORC1) to the lysosome membrane where it becomes activated, resulting in the activation of the downstream anabolic signalling pathways that control muscle tissue protein synthesis [59–61]. The current leucine requirement within a given protein source is set at 5.9% by the WHO/FAO/UNU [58]. Whereas plant-based proteins like hemp (5.1% leucine) and lupin (5.2%) fall short, other proteins like oat (5.9%), spirulina (6.0%) and wheat (6.1%) protein provide close to the recommended leucine content. Moreover, plant-based proteins like soy (6.9%), canola (6.9%), pea (7.2%), brown rice (7.4%), potato (8.3%) and corn (13.5%) protein have leucine contents that exceed the recommended requirements. The leucine content of potato protein (8.3%) is even higher when compared with casein (8.0%) or egg (7.0%) protein. Furthermore, the leucine content of corn protein (13.5%) is even higher than whey protein (11.0%), the latter of which is typically regarded as the protein with the highest leucine content and the strongest anabolic potential among the animal-derived proteins (Fig. 1b).

Previous studies have shown that ingesting 20–25 g whey protein (providing 2.2–2.7 g leucine) strongly increases muscle protein synthesis rates [11, 62–64]. The amount of ingested leucine required to maximally stimulate the muscle protein synthetic machinery may be modulated by its protein matrix (e.g. digestion and absorption kinetics, and availability of other amino acids). However, if we assume that ingestion of 2.7 g leucine is sufficient to maximally trigger the muscle protein synthetic machinery, it is evident that this can also be achieved by the ingestion of plant-based protein sources. Plant-based proteins may provide the same amount of leucine simply by providing an equivalent amount of protein based on their intrinsic leucine content. For example, for corn-derived protein (13.5% leucine), ingestion of merely 20 g protein would already provide 2.7 g of leucine. In contrast, > 25 g of other plant-based proteins would have to be ingested to deliver 2.7 g leucine. In fact, ingestion of ~ 33 g potato, ~ 37 g brown rice, ~ 38 g pea, ~ 40 g canola, ~ 40 g soy, and ~ 45 g wheat protein would be required to ingest 2.7 g of leucine [39]. From the analysed proteins and protein sources (Fig. 1b), quinoa protein seems to have the lowest leucine content (3.8%). It would require ~ 71 g quinoa protein to be ingested to provide 2.7 g leucine. Of course, this only represents the amount of leucine believed to fully activate the muscle protein synthetic machinery. Besides activating the signalling pathways that stimulate muscle protein synthesis, ample essential amino acids may be required as precursors to allow efficient muscle protein accretion [65]. An insufficient provision of one (or more) essential or non-essential amino acids would theoretically be restrictive and, as such, attenuate the post-prandial rise in muscle protein synthesis rate.

Besides having a relatively low essential amino acid content (i.e. low leucine content), many plant-based proteins are deficient in one or more specific amino acid. Plant-based proteins are often particularly low in lysine and/or methionine content (ranging from 1.4 to 6% and 0.2 to 2.5%, respectively) when compared with animal-based proteins (ranging from 5.3 to 9.0% and 2.2 to 2.8%, respectively; Fig. 1c, d). The lysine content of wheat (1.4%), corn (1.5%), oat (2.1%), brown rice (2.4%), pumpkin seeds (2.7%), sunflower seeds (2.8%), hemp (2.8%), quinoa (3.3%), spirulina (3.5%), and lupin (3.5%) protein are well below the WHO/FAO/UNU requirements (4.5%) and substantially lower when compared with soy (4.6%), canola (5.9%), pea (5.9%), and potato (6.0%) protein (Fig. 1c). A considerable number of plant-based proteins also fall short for methionine requirements (1.6%), with oat (0.2%), field bean (0.2%), brown bean (0.3%), lentil (0.3%), chickpea (0.3%), marrowfat pea (0.3%), lupin (0.3%), pea (0.4%), soy (0.4%), quinoa (0.6%), and wheat (0.9%) protein providing much less methionine. In contrast, other plant-based proteins such as potato (1.6%), corn (1.7%), spirulina (1.7%), sunflower seed (1.7%), pumpkin seed (1.9%), hemp (2.0%), canola (2.2%), and brown rice (2.5%) protein tend to meet the methionine content requirements (Fig. 1d). Clearly, there is considerable variability in amino acid composition between the many different plant-based proteins and plant-based protein sources.

Only a handful of studies have directly compared post-prandial muscle protein synthesis rates following ingestion of plant- versus animal-derived proteins [13, 14, 16, 36–38]. Ingestion of soy protein has been shown to be less effective in stimulating post-prandial muscle protein synthesis rates when compared with the ingestion of an equivalent amount of whey protein in both young and older adults at rest and during recovery from exercise [13, 14, 36], but more effective than casein protein [13]. Furthermore, Yang et al. [14] showed that ingesting a greater amount (40 g vs 20 g) of soy protein did not compensate for the lesser muscle protein synthetic response when compared with the ingestion of 20 g whey protein isolate. We observed no significant post-prandial increase in muscle protein synthesis rates following the ingestion of 35 g wheat protein hydrolysate in a group of healthy older men [16]. When we increased the amount of wheat protein hydrolysate to 60 g, thereby providing the same amount of leucine as provided in 35 g whey protein, we observed a robust increase in muscle protein synthesis rates. Clearly, these data seem to support the hypothesis that differences in amino acid composition can be, at least partly, compensated for by ingesting greater amounts of the specific protein source.

More recently, we observed no differences in post-prandial muscle protein synthesis rates following the ingestion of 30 g wheat protein hydrolysate or the same amount of milk protein concentrate [38]. In contrast to the earlier work in our group, this study was performed in young, recreationally active adults. The greater sensitivity of skeletal muscle tissue to the anabolic properties of amino acids due to the higher habitual activity level in younger, more active adults [66, 67] may have been responsible for the absence of any measurable differences in the post-prandial muscle protein synthetic response to the ingestion of 30 g wheat- versus milk-derived protein. Clearly, we need to understand that differences in the anabolic responses to the ingestion of plant versus animal-based protein sources will also depend on the amount of protein provided and the specific population in which the comparison is made.

In short, the amino acid composition of plant-based protein sources can be highly variable. Therefore, more studies are warranted to assess the anabolic properties of various plant- and animal-derived proteins and protein sources beyond the few comparisons that are currently available (soy and wheat protein). Furthermore, it should be noted that the outcome of these comparisons will likely differ depending on the amount of protein ingested and the population and setting in which the comparisons are being made.

## Improving the Anabolic Properties of Plant-Based Proteins

**Fig. 2:**
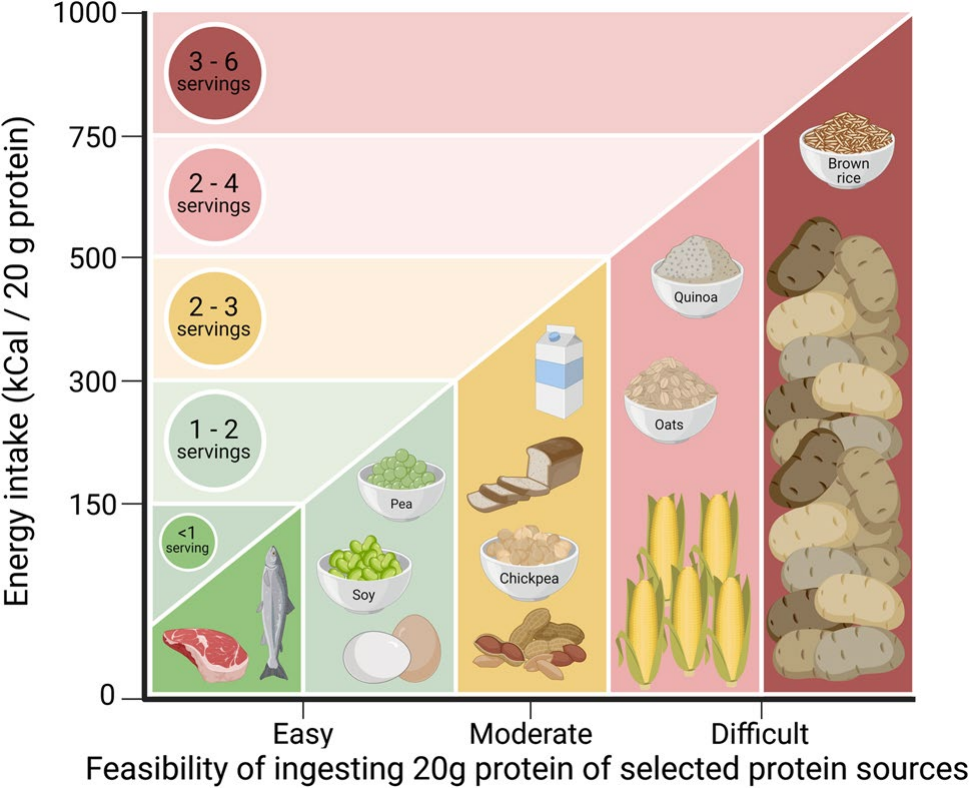
Categorical representation of the feasibility of consuming 20 g protein provided by ingesting the whole food source (*x*-axis), with the amount of food that needs to be consumed expressed as servings with the concomitant energy intake equivalent (*y*-axis). Serving sizes: meat/salmon: ~ 100 g, egg: ~ 120 g (2 eggs), soy: ~ 100 g, pea: ~ 150 g, chickpea: ~ 150 g, peanut: ~ 50 g, bread (wheat): ~ 70 g (2 slices), milk: ~ 200 mL, corn: ~ 150 g, oats ~ 40 g (raw), quinoa: ~ 75 g (raw), brown rice: ~ 75 g (raw), potato: 175 g

As discussed previously, the proposed lesser anabolic properties of plant-based versus animal-based proteins may be attributed to differences in protein absorbability, protein digestion and amino acid absorption kinetics, and/or amino acid composition of the proteins. There are various nutritional strategies that may be applied to improve the anabolic properties of plant-based proteins depending on the factor(s) responsible for the proposed lower anabolic capacity (Fig. 2).

The absorbability of a plant-based protein source is often compromised by the presence of anti-nutritional factors in plant-based protein sources, such as fibre and polyphenolic tannins [43]. Processing of whole foods can strongly increase the absorbability of intrinsic protein. Dehulling of beans prior to consumption has been shown to represent an effective means to increase the capacity to absorb the intrinsic protein [44]. Extraction of protein and purification from anti-nutritional factors to produce a plant-derived protein isolate or concentrate further improves the efficiency by which plant-based proteins can be absorbed [45]. Furthermore, heat treatment and hydrolysation of the protein further increase digestibility and/or improve protein digestion and amino acid absorption kinetics [3, 68]. These processes are typically applied in most plant- as well as animal-based protein sources that we purchase either as (processed) food products or as protein isolates or concentrates. Clearly, when dealing with foods the various processes involved in harvest, processing, storage, cooking, chewing and ingestion all contribute to the absorbability of the final protein source and the rate of its protein digestion and amino acid absorption. These processes also differ between the various foods that together form our composite meals. Future work will need to address the anabolic properties of actual foods and, more importantly, the muscle protein synthetic response to the ingestion of complete meals.

**Fig. 3:**
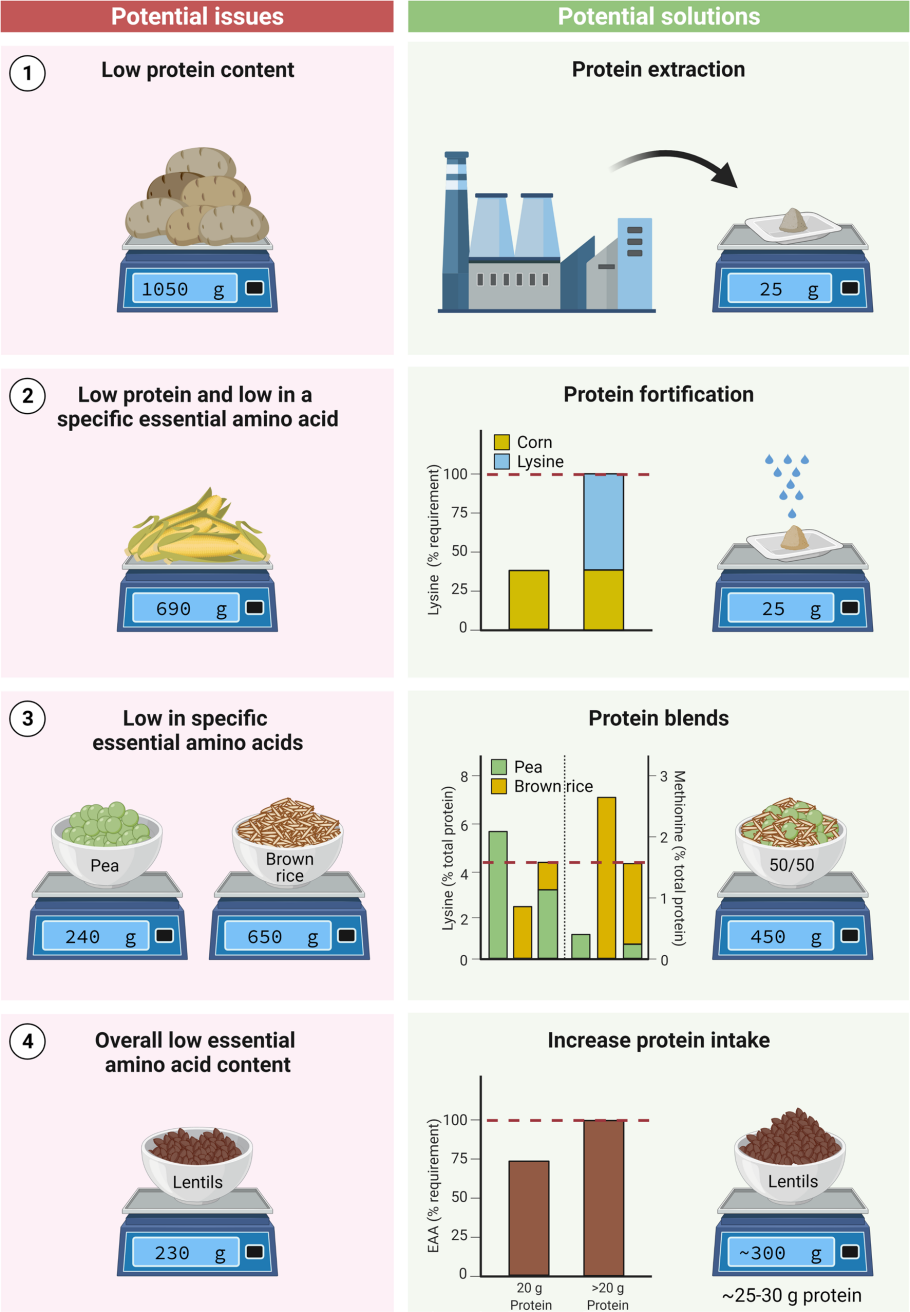
Overview of potential issues and solutions to optimise the anabolic response following plant-based protein consumption. (1) For plant-based foods with a high protein quality, but low protein content (e.g. potato), extraction of high-quality protein isolates forms an effective method to allow ingestion of a desired amount of protein. (2) For plant-based food sources with deficiencies in specific amino acids (e.g. corn: low in lysine), a protein isolate or concentrate can be fortified with the deficient free amino acid(s) to improve the amino acid content profile. (3) Plant-based food sources with deficiencies in specific essential amino acids can be combined to improve the overall amino acid profile of the protein blend. For example, peas are low in methionine but high in lysine; in contrast, brown rice is high in methionine but low in lysine. A blend combining pea and brown rice would meet overall amino acid requirements. (4) When plant-based food sources (or protein isolates) are deficient in one or more amino acids (e.g. lentils, wheat), this may be compensated for by simply ingesting a greater amount of the plant-based protein source. Illustrations: the scale balance represents the amount of food to be consumed to provide 20 g protein, unless otherwise indicated. Weight for brown rice and lentils represent cooked amounts. Dashed horizontal line in graphs represents the amino acid requirements for adults (WHO/FAO/UNU Expert Consultation 2007 [58]). *EAA* Essential amino acid

**Fig. 4:**
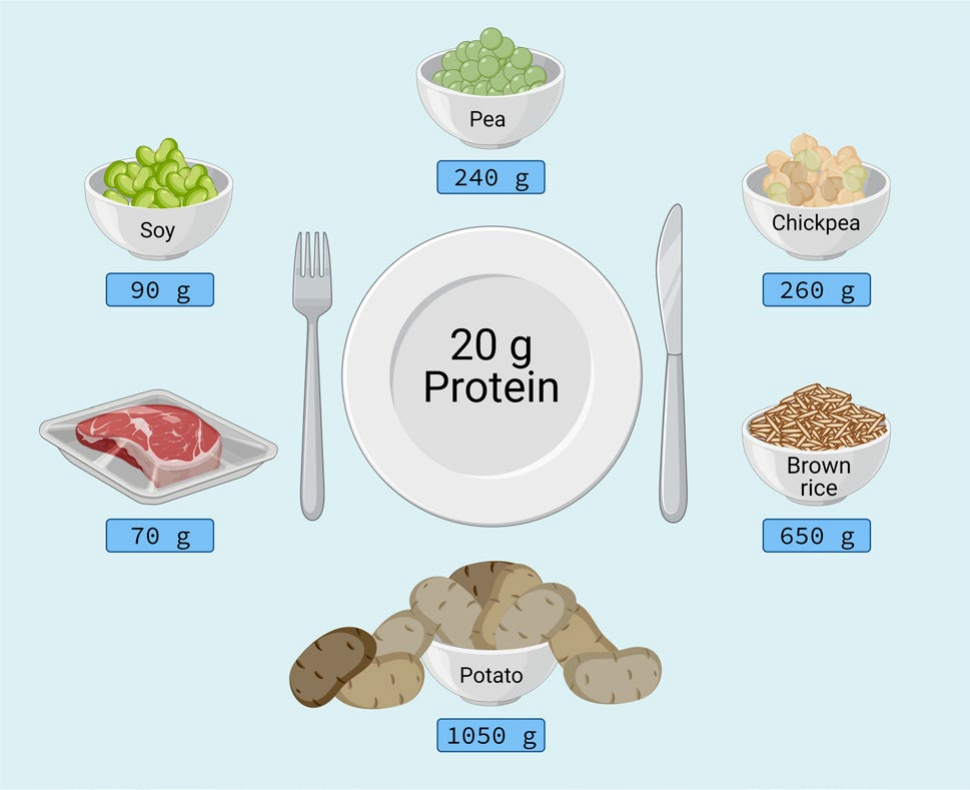
Amount of the selected whole-food protein sources to be consumed to allow ingestion of 20 g protein. Illustrated are meat, soy, pea, chickpea, brown rice and potato in order of protein content (from high to low)

The lesser anabolic properties of some plant-based proteins may be attributed to the low(er) essential amino acid content and/or specific amino acid deficiencies of that protein. The easiest way to compensate for the lower protein quality of a plant-based versus animal-based protein source is to simply consume a greater amount of the lesser quality protein (Fig. 2). In support, we observed that ingestion of 60 g as opposed to 35 g of a wheat protein hydrolysate effectively increased post-prandial muscle protein synthesis rates in a group of healthy older men [16]. Although this strategy may not apply to all plant-based proteins [64], increasing the protein dosage to compensate for either the lower essential amino content or a specific amino acid deficiency should theoretically improve the post-prandial protein synthetic response. However, while such a strategy would be easy to apply when considering the use of a plant-derived protein isolate or concentrate, it may not always be practical or feasible when considering plant-based (whole) foods. The lower protein density of most plant-based protein sources would greatly increase both the total caloric content and volume of the plant-based food that would need to be consumed. Simply consuming 20 g protein in the form of a plant-based protein source is already challenging, both from a perspective of food volume as well as caloric content (Figs. 3, 4). Current research has focused on evaluating the anabolic properties of plant-based protein isolates or hydrolysates. Ingesting ample amounts of a single plant-based protein in the form of its whole food will not always be feasible, especially in a more clinical setting in which food intake is generally compromised, or in a sport setting where athletes need to adhere to strict caloric intakes.

An alternative strategy to increase the anabolic potential of a plant-based protein is to combine different protein types and/or sources to provide a protein blend with a more balanced amino acid profile. Whereas some plant-based proteins are particularly deficient in lysine, others are deficient in methionine [39]. For example, corn, hemp, brown rice, soy and pea protein are low in lysine and/or methionine content. For each protein source, this deficiency could be compensated for by consuming 2–4 times more of the same protein. However, combining corn, hemp, or brown rice protein (low lysine and high methionine content) with an equal amount of soy or pea protein (low methionine and high lysine content) provides a blend with a more balanced amino acid profile (Fig. 2). Such blends would require only 1.1–1.9 times more protein to be consumed to compensate for specific amino acid deficiencies [39]. Besides exclusive plant-based protein blends, combinations of plant- plus animal-derived proteins may also play an important role in the trend to lower animal-derived food consumption without compromising protein quality. Oat, lupin, quinoa, and wheat protein are low in both lysine and methionine content, which could theoretically be compensated for by ingesting 3–8 times more of the respective protein. However, blending these proteins with an equal amount of an animal-derived protein would require only 1.05–1.4 times more of the respective protein blend to be consumed to provide sufficient amounts of all essential amino acids [39]. Such protein blends would represent the composition of an omnivorous diet, in which ~ 40–50% of the consumed protein is generally derived from plant-based sources [69]. In support, robust increases in post-prandial muscle protein synthesis rates have been reported following ingestion of whey, casein and soy protein blends [70–72]. More recently, we observed no differences in the post-prandial muscle protein synthetic response following ingestion of 30 g milk or a 30 g protein blend combining wheat and milk protein [38]. Many more protein blends combining two or more protein sources in various ratios can be composed to achieve particular aims regarding amino acid composition, price, taste, and sustainability without compromising the capacity to stimulate muscle protein synthesis.

If a specific amino acid deficiency forms the limiting factor for a plant-based protein to increase post-prandial muscle protein synthesis rates, an alternative option would be to fortify the protein with one or more specific (free) amino acids. As leucine is considered to be fundamental to the post-prandial muscle protein synthetic response, fortification with free leucine could represent a feasible strategy to augment post-prandial muscle protein synthesis rates. In support, leucine fortification of a bolus of intact protein, amino acid mixture, or mixed meal has been reported to further increase post-prandial muscle protein synthesis rates [18, 20, 73, 74]. To our knowledge, there are not many data available on the impact of leucine fortification of plant-based proteins on subsequent post-prandial muscle protein synthesis rates. A study in rodents demonstrated lower muscle protein synthesis rates after feeding with wheat versus whey protein [75]. Fortification of the wheat protein with free leucine, to match the leucine content in an equivalent amount of whey protein, increased muscle protein synthesis rates to a level that was no longer different from the response observed after whey protein feeding. In contrast, we did not observe higher post-prandial muscle protein synthesis rates following ingestion of 20 g soy protein fortified with 2.5 g free leucine compared with 20 g soy protein only during recovery from exercise in young adults [37]. In fact, we observed no measurable differences in post-prandial muscle protein synthesis rates following ingestion of 20 g whey, 20 soy, or 20 g soy fortified with 2.5 g free leucine to match the amount of leucine present in 20 g whey [14]. We can only assume that under these conditions the leucine content was not a limiting factor to the post-prandial rise in muscle protein synthesis rates. This may be explained by the exercise-induced increase in skeletal muscle tissue sensitivity to the stimulating properties of an increase in circulating leucine concentration. With many plant-based proteins being deficient in lysine and/or methionine, it has been hypothesised that fortification of these plant-based proteins with their respective deficient amino acid(s) may amplify their anabolic potential (Fig. 2). Although fortification with selected free amino acids is commonly applied in plant-based products designed to replace meat or dairy products, there are no studies that have assessed the efficacy of such a strategy as a means to improve the anabolic properties of plant-based protein ingestion.

## Post-Prandial Protein Handling Following Meal Ingestion

Work on the anabolic properties of plant-based proteins has been largely confined to the comparison of post-prandial muscle protein synthesis rates following ingestion of a handful of plant- versus animal-derived protein isolates or concentrates. However, dietary protein is generally consumed in the form of a whole food or food product and as part of a more complete, composite meal. This automatically provides a blend of different plant-based protein sources, improving the post-prandial muscle protein synthetic response. Furthermore, when consuming protein as part of a product and/or meal, other nutrients such as carbohydrates, fats, micronutrients, and other (anti-) nutritional compounds may modify post-prandial protein digestion and amino acid absorption kinetics and subsequent muscle protein synthesis rates [76]. In support, we [77–79] have shown that post-prandial protein digestion and amino acid absorption may be delayed when carbohydrate or fat are co-ingested with protein. However, this does not seem to have much impact on post-prandial muscle protein synthesis rates [77, 80]. In addition, it has been suggested that co-ingestion of carbohydrate with protein could increase post-prandial muscle protein synthesis rates by stimulating post-prandial insulin release. However, the impact of endogenous insulin release on post-prandial muscle protein synthesis rate has proven permissive rather than stimulatory and the modest increase in insulin release observed following protein ingestion only is already sufficient to allow post-prandial muscle protein synthesis to reach maximal values [81]. In support, co-ingestion of carbohydrate with protein has been proven not to augment post-prandial muscle protein synthesis rates either at rest [77, 78, 82] or during recovery from exercise [79, 83, 84].

Although such studies provide insight into the impact of co-ingesting other macronutrients on protein digestion and amino acid absorption kinetics and the subsequent post-prandial stimulation of muscle protein synthesis, they do not necessarily reflect the anabolic response to the ingestion of the whole foods from which they are derived. Whereas several studies have assessed post-prandial muscle protein synthesis rates following the ingestion of whole foods such as milk [32], meat [10, 32–34], and eggs [85], there are fewer data available on the anabolic responses to the ingestion of plant-based whole foods. This knowledge gap prevents us from understanding the true anabolic properties of consuming plant-based foods as the food matrix of plant-based foods may compromise protein digestion and amino acid absorption kinetics and, as such, attenuate the postprandial rise in muscle protein synthesis rates. Previous work has shown substantial differences in post-prandial plasma amino acid responses following ingestion of an egg- versus cereal-based breakfast, providing an isonitrogenous amount of protein [86]. The observed differences in the post-prandial rise in plasma amino acid concentrations following the egg- versus cereal-based breakfast did not result in differences in muscle protein synthesis rates. This clearly shows that the muscle protein synthetic response to meal ingestion is complex and can not be predicted by simply assessing protein amino acid composition or post-prandial plasma amino acid profiles.

The matrix of whole foods, food products and/or composite meals is, at least partly, defined by the combination of a variety of macronutrients, micronutrients, and (anti-)nutritional compounds. However, the food matrix is also modified by commercial food processing as well as in-house food preparation, which often includes heating and/or cooking [3, 87, 88]. Prior to consumption, food is cut or mashed and chewed, which will also impact the rate of protein digestion and amino acid absorption [3, 89, 90]. Numerous factors play a role in determining the post-prandial muscle protein synthetic response to food ingestion. Besides the impact of individual food matrices on protein digestion and amino acid absorption kinetics, it is important to consider that a composite meal often includes a variety of animal and plant-based foods, or at least various plant-based foods. There is currently limited information within the literature on the (potential) interaction between different protein sources within a single meal on protein digestion and amino acid absorption kinetics and the post-prandial muscle protein synthetic response to feeding.

Although we have gained much insight into the various factors that modulate dietary protein absorbability, protein digestion and amino acid absorption, and post-prandial muscle protein synthesis, we lack insights into post-prandial protein handling following ingestion of whole foods and mixed meals. Future studies are warranted to assess the anabolic properties of composite meal ingestion and the impact this can have on muscle conditioning in both health and disease.

## Plant-Based Proteins in Sports Nutrition

The transition towards a more plant-based diet has attracted much interest among athletes. Not surprisingly, this also raises questions regarding the impact of the (lower) quality of plant-based proteins on muscle conditioning during recovery from exercise. There are only a handful studies that have compared post-exercise muscle protein synthetic responses following the ingestion of plant- versus animal-derived proteins [13, 14, 16, 36–38]. In these studies, the main plant-derived protein that has been applied is soy protein. Some [13, 14, 36], but certainly not all [37], studies have reported less of an increase in post-exercise muscle protein synthesis rates following ingestion of soy protein when compared with an equivalent amount of milk or whey protein. Furthermore, soy protein has been shown to result in greater muscle protein synthesis rates during 3 h of post-exercise recovery when compared with casein protein [13]. As exercise makes the muscle more sensitive to the anabolic properties of amino acid or protein administration, it could be speculated that the post-prandial rise in circulating plasma leucine concentration is of lesser importance when protein is consumed following exercise. Therefore, the lower leucine content of most plant-based proteins may no longer restrict post-prandial muscle protein synthesis rates during recovery from exercise. Consequently, the capacity of a protein to stimulate post-exercise muscle protein synthesis is more likely to be determined by the amount of amino acids provided as precursors for protein synthesis. Therefore, an ample provision of all amino acids without deficiencies in specific amino acids may be of primary importance when determining the optimal plant-based protein (blend) to support post-exercise muscle conditioning. Clearly, research is warranted to compare muscle protein synthesis rates during recovery from exercise while ingesting different plant- versus animal-based proteins or protein sources. Those studies will provide insight into the preferred characteristics of a dietary protein (blend) that would optimise the skeletal muscle adaptive response to exercise.

Longer-term intervention studies assessing the impact of protein supplementation on the adaptive response to resistance-type exercise training tend to show greater gains in muscle mass and strength when applying protein supplementation [91, 92]. Increases in daily muscle protein synthesis rates and/or gains in muscle mass have been reported following resistance-type exercise training while supplementing plant-derived protein sources, such as soy [93–96], pea [97], rice [98] and potato [99] protein. However, whether these gains in muscle mass and strength during resistance-type exercise training differ from the gains observed when an equivalent amount of animal-based protein is supplemented remains equivocal. A recent meta-analysis concluded that the animal- or plant-based origin of the supplemented protein source does not impact the gains in lean mass or muscle strength following prolonged resistance-type exercise training [100]. However, it seems evident that this conclusion would depend also on the population, the type of training, the training status of the volunteers, and most of all the amount of protein supplemented and the overall habitual protein intake. Recent work by Hevia-Larraín et al. [101] reported no differences in muscle mass and strength accrual following prolonged resistance exercise training while consuming either an exclusively plant-based or an omnivorous diet. This may not be too much of a surprise as the untrained subjects were consuming a high-protein intake diet (~ 1.6 g/kg body mass/day) throughout the exercise intervention period, with substantial amounts of protein (soy or whey protein isolates) being supplemented twice daily.

Based upon the described differences in protein absorbability, protein digestion and amino acid kinetics, and post-prandial muscle protein synthesis rates following ingestion of plant- versus animal-based protein sources, we could hypothesise that when transitioning towards a more plant-based diet, more dietary protein would be required to allow the same stimulation of muscle protein synthesis rates. This would also imply that more plant-based proteins should be consumed and/or supplemented to achieve the same level of muscle mass accretion during prolonged resistance-type exercise training. However, most athletes already consume ample amounts of protein due to their higher energy intake. A nation-wide survey of well-trained athletes reported a protein intake of ~ 1.5 g protein per kg body mass per day [102]. Although this represents a daily protein intake well above the Recommended Daily Allowance (RDA) proposed by the WHO (0.8 g/kg/day), it has been argued that a protein intake of 1.6 g/kg would maximise gains in muscle mass and strength during prolonged resistance-type exercise training [92]. Consequently, it could be speculated that a diet providing low(er) quality protein could compromise the skeletal muscle adaptive response to exercise training. However, the latter represents more an academic concept as small differences in protein quality will not have much impact on the adaptive response to exercise training when such large amounts of protein are habitually consumed. Furthermore, omnivorous athletes already derive > 40% of their habitual daily protein intake from plant-based sources [102].

More important is the potential negative impact of a transition towards a more plant-based diet in conditions where athletes lower their energy intake and, as such, reduce protein consumption. Athletes trying to reduce body weight by caloric restriction or athletes recovering from an injury would actually require a similar or even higher (absolute) protein intake while consuming less food. In such conditions the quality of the consumed protein is of the utmost importance, and transitioning to a diet with less anabolic properties could compromise muscle maintenance or attenuate muscle regain. Therefore, we need to evaluate the positive as well as the potentially negative aspects of transitioning towards a more plant-based diet. Furthermore, we need to evaluate whether this is accompanied by a transition towards greater dietary protein intake requirements. Work is needed to evaluate the impact of structurally consuming a more exclusive plant-based whole-foods diet on muscle mass and function in various populations, in both health and disease.

## Alternative Protein Sources

Huge investments are presently being made in the search for more sustainable production of high-quality protein sources that are not derived from animals. This process has now expanded from plant-based protein sources to various other protein sources, including the growing of yeast, fungi, micro-algae, the breeding of insects, and even the cultivation of lab-grown meat as potential protein sources for human consumption. Although a discussion on these alternative, sustainable protein sources is beyond the scope of this review, we will address two of these protein sources as they have recently been assessed for their capacity to stimulate post-prandial muscle protein synthesis rates in vivo in humans.

Recent work has addressed the anabolic properties of a food source derived from cultivating a fungus (*Fusarium venenatum*), resulting in what has been coined mycoprotein [103–105]. This protein source has been reported to have a high protein content (~ 45%) with the protein showing an amino acid composition that does not differ much from dairy protein [106]. Prior work suggested good digestibility based upon the observation that post-prandial plasma essential amino acid (and leucine) concentrations were comparable following ingestion of mycoprotein when compared with the ingestion of an equivalent amount of milk protein. More recently, these investigators followed up by showing that ingestion of a single bolus of mycoprotein (70 g, providing 31.5 g protein) increased both resting and post-exercise muscle protein synthesis rates in young males, with a post-prandial muscle protein synthetic response that was greater than the response observed after ingesting a leucine-matched bolus of milk protein (31 g, providing 26.2 g protein) [103]. These data show that fungi can provide a viable, high-quality protein source that is effective in stimulating muscle protein synthesis.

Another alternative dietary protein source that has attracted much interest is edible insects. Although technically insects also classify as animals, they can be produced on a more viable and sustainable commercial scale and, as such, they form another promising candidate to contribute to ensuring global food security [107, 108]. Insects have a high protein content and their protein has an amino acid composition that closely resembles conventional high-quality animal-derived proteins [107]. Recently, we produced intrinsically labelled lesser mealworms by feeding these larvae with stable isotope labelled amino acids [25], allowing us to directly quantitate protein digestion and amino acid absorption kinetics and the subsequent muscle protein synthetic response at rest and during recovery from exercise following ingestion of a single bolus of mealworms. The mealworm-derived protein was rapidly digested and absorbed and strongly increased post-prandial muscle protein synthesis rates. In fact, the observed post-prandial muscle protein synthetic response did not differ from the response observed after the ingestion of an equivalent amount of milk protein [109].

These are just two examples of other alternative, high-quality protein sources that can be produced on a viable and more sustainable commercial scale and that seem to have anabolic properties that do not differ from the conventional animal-based protein sources. Clearly, more work will be performed to establish the digestion and absorption kinetics of many of these novel protein sources and evaluate their post-prandial anabolic properties. There seem to be many opportunities for the production of alternative protein sources to successfully meet future global dietary protein demands.

## Conclusions

There is a global trend of a transition towards the consumption of a more plant-based diet. Ingestion of plant-derived proteins is generally considered to result in lower post-prandial muscle protein synthesis responses when compared with the ingestion of an equivalent amount of animal-derived protein. The lesser anabolic properties of plant-based versus animal-derived proteins have been attributed to differences in their protein digestion and amino acid absorption kinetics and amino acid composition. Most plant-based proteins have a low(er) essential amino acid content and are often deficient in one or more specific amino acids, such as lysine and methionine. However, there are large differences in amino acid composition between various plant-derived proteins or plant-based protein sources. So far, only a few studies have directly compared the muscle protein synthetic response following the ingestion of a plant- versus animal-derived protein. The proposed lower anabolic properties of plant- versus animal-derived proteins may be compensated for by (i) consuming a greater amount of the plant-derived protein or plant-based protein source to compensate for the lesser quality; (ii) using specific blends of plant-derived proteins to create a more balanced amino acid profile; or (iii) fortifying the plant-based protein (source) with the specific free amino acid(s) that is (are) deficient. Clinical studies are warranted to assess the anabolic properties of the various plant-based proteins and their protein sources and to identify the factors that may or may not compromise the capacity to stimulate post-prandial muscle protein synthesis rates in vivo in humans. Healthy, active athletes typically consume a diet that provides well above ~ 1.5 g protein per day. The consumption of more plant-based protein(s) should, therefore, not necessarily lead to a less than optimal protein intake. Accordingly, there are ample data to show that protein supplementation with plant-derived proteins can (also) support greater gains in muscle mass and strength when combined with prolonged resistance-type exercise training. Under conditions of low energy intake, as observed during dietary interventions to support body fat loss or in clinically compromised patients, it could be speculated that transition towards a more plant-based diet could compromise the post-prandial stimulation of muscle protein synthesis rates. Consequently, future work will need to establish whether the transition towards a more exclusive plant-based diet is accompanied by a transition towards greater dietary protein intake requirements.

## Declarations

### Figures

Figures were created with Biorender.com.
